# Molecular Characterization of Two Monoclonal Antibodies against the Same Epitope on B-Cell Receptor Associated Protein 31

**DOI:** 10.1371/journal.pone.0167527

**Published:** 2016-12-01

**Authors:** Won-Tae Kim, Saemina Shin, Hyo Jeong Hwang, Min Kyu Kim, Han-Sung Jung, Hwangseo Park, Chun Jeih Ryu

**Affiliations:** 1 Institute of Anticancer Medicine Development, Department of Integrative Bioscience and Biotechnology, Sejong University, Seoul, Korea; 2 Division in Anatomy and Developmental Biology, Department of Oral Biology, Oral Science Research Center, BK21 PLUS Project, Yonsei University College of Dentistry, Seoul, Korea; 3 Oral Biosciences, Faculty of Dentistry, The University of Hong Kong, Hong Kong SAR; University of Edinburgh, UNITED KINGDOM

## Abstract

Previously, we showed that B-cell receptor associated protein 31 (BAP31), an endoplasmic reticulum (ER) membrane chaperone, is also expressed on the cell surface by two monoclonal antibodies (MAbs) 297-D4 and 144-A8. Both MAbs recognize the same linear epitope on the C-terminal domain of BAP31, although they were independently established. Here, flow cytometric analysis showed that 144-A8 had additional binding properties to some cells, as compared to 297-D4. Quantitative antigen binding assays also showed that 144-A8 had higher antigen binding capacity than 297-D4. Affinity measurement revealed that 144-A8 had 1.54-fold higher binding affinity than 297-D4. Analysis of the heavy- and light-chain variable region sequences of two MAbs revealed that both MAbs belonged to the same heavy chain (Igh-V3660 VH3) and light chain subgroup (IGKV21) with just two amino acid differences in each framework region, indicating that both MAbs arise from the same germline origin. Seven amino acid differences were found between the complementarity determining regions (CDRs) of the two MAbs. Molecular modeling of the epitope-paratope complexes revealed that the epitope appeared to reside in closer proximity to the CDRs of 144-A8 than to those of 297-D4 with the stronger hydrogen bond interactions with the former than the latter. More interestingly, an additional hydrophobic interaction appeared to be established between the leucine residue of epitope and the paratope of 144-A8, due to the substitution of H-Tyr101 for H-Phe101 in 144-A8. Thus, the different binding specificity and affinity of 144-A8 appeared to be due to the different hydrogen bonds and hydrophobic interaction induced by the alterations of amino acids in CDRs of 144-A8. The results provide molecular insights into how the binding specificities and affinities of antibodies evolve with the same epitope in different microenvironments.

## Introduction

B-cell receptor associated protein 31 (BAP31) is a 28 kDa integral endoplasmic reticulum (ER) membrane protein and expressed ubiquitously [1–3]. BAP31 is composed of three membrane-spanning fragments and 13 kDa of the cytoplasmic tail. BAP31 also promotes the vesicular transport of transmembrane proteins, such as class I major histocompatibility complex [4, 5], cellubrevin [6], membrane-bound immunoglobulin D [7], and leukocyte integrin CD11b/CD18 [8], by associating with transport complexes. Thus, BAP31 regulates the fate of integral ER membrane proteins as a molecular chaperone and a quality control factor [9]. BAP31 is also an important factor of apoptosis because it interacts with Bcl-2/Bcl-xL and procaspase-8L on the ER membrane [3, 10]. BAP31 is also associated with complex crosstalk between the two organelles during apoptosis, by interaction between ER-localized BAP31 and Fis1 at the mitochondrial outer membrane [11, 12].

Previously, we generated monoclonal antibodies (MAbs) against surface molecules of undifferentiated human embryonic stem cells (hESCs) by using modified decoy immunization strategy [13]. Among the MAbs, 297-D4 recognizes BAP31 on the surface of hESCs, which regulates hESC adhesion, stemness, and survival by interacting with epithelial cell adhesion molecule (EpCAM) [14]. A subsequent study found that 144-A8, an independently isolated MAb, also recognizes cell surface-expressed BAP31, and both MAbs recognize the same epitope, which is mapped to the residues 208–217 of BAP31 [15]. The present study found that both MAbs showed different binding patterns in flow cytometric analyses and quantitative binding studies, although both recognized the same epitope on BAP31. Affinity measurement of two MAbs showed that the affinity of 144-A8 for recombinant BAP31 was substantially higher than that of 297-D4. Therefore, we cloned and sequenced the immunoglobulin heavy- and light-chain variable region sequences of the two MAbs and found seven amino acid differences between the CDRs of 144-A8 and 297-D4. To further elucidate the molecular mechanism of higher affinity of 144-A8 against the epitope, molecular modeling combined with molecular docking of the two epitope-paratope complexes was performed and compared.

## Materials and Methods

### Purification of antibodies and GST-BAP31 fusion protein

MAbs were purified from the culture supernatant of hybridoma by Protein G-Sepharose column chromatography, as described previously [14]. BAP31 was expressed as a fusion protein with glutathione-S-transferase (GST) in *E*. *coli*. To prevent the formation of the insoluble inclusion body, the C-terminal domain (residues 124–246) of BAP31, transmembrane domain-free BAP31 fragment, was subcloned into the EcoRI/SalI sites of pGEX4T-2 (GE Healthcare, Seoul, Korea). The expression of the fusion protein was induced by 0.1 mM isoprophyl-β-D-thiogalactopyranoside at 32°C for 6 h and purified by chromatography on the glutathione Sepharose column, as described in the previous study [15]. The protein concentration was measured by bicinchoninic assay (Thermos Scientific, Seoul, Korea). The purified proteins were subjected to 12% SDS-PAGE, stained with Coomassie Brilliant Blue R-250, and analyzed by western blot analysis.

### Indirect enzyme-linked immunosorbent assay (ELISA)

To measure the antigen binding ability of the two MAbs, 96-well microtiter plates were coated with 20 μg/ml of purified antigen in 100 μl of coating buffer (50 mM sodium carbonate, 50 mM sodium bicarbonate, pH 9.6) at 4°C overnight and blocked with 5% skim milk. After washing with phosphate-buffered saline containing 0.05% Tween-20 (PBST), the plates were incubated with serial dilutions (0, 0.02, 0.04, 0.1, 0.5, 1, 2, and 4 μg/ml) of antibodies at 37°C for 1 h. After washing with PBST, the plates were further incubated with horse radish peroxidase-conjugated anti-mouse IgG antibody (Sigma-Aldrich, St. Louis, MO, USA) at 37°C for 1 h. Each well was then incubated with PC buffer (0.2 M citrate-PO_4_, pH 5.0) containing 0.04% o-phenylenediamine and 0.03% H_2_O_2_ for 20 min. The reaction was stopped by 2 M of H_2_SO_4_, and the optical density was measured at 490 nm in an ELISA reader. To determine concentration of antigen, 96-well microtiter plates were coated with serial dilutions (0, 0.1, 0.2, 0.5, 1, 10, 20 μg/ml) of antigen in coating buffer at 4°C overnight. After blocking with 5% skim milk, each well was incubated with 0.04 μg/ml of antibody at 4°C for 1 h. Further experimental steps were the same, as described above.

To determine the binding affinity of the monoclonal antibodies, a solution containing 100 ng of the antibodies and various concentrations of GST-fused BAP31 protein (10^−12^–10^−6^ M) were pre-incubated at 37°C for 2 h and added to the ELISA plate coated with 20 μg/ml of the same antigen. The concentrations of free antibody were determined by the same procedure, as described above. The antibody affinity was estimated as the interaction of the antigen concentration required to inhibit 50% maximal binding by competitive ELISA [16].

### Heavy and light chain cDNA cloning and sequencing

Total RNAs were isolated from hybridomas using RNeasy mini kit (Qiagen, Gaithersburg, MD, USA), according to the manufacturer’s recommendation. Heavy and light chain cDNAs were generated from RNAs by using One-Step RT-PCR PreMix Kit (iNtRON Biotechnology, Seoul, Korea), according to the manufacturer’s protocol. The coding regions were amplified by polymerase chain reaction (PCR) using specific primers, described as a previous study [17]. The amplified cDNAs were cloned into pBluescript cloning vector using the EcoRI/SalI or HindIII/SalI restriction enzyme sites. Each DNA was used to transform DH5α bacterial cells and sequenced using the M13 primers. Complementarity determining regions (CDRs) of antibodies were defined from a comparison of all known antibody sequences [18].

### Molecular modeling studies of binding of BAP31 epitope with 144-A8 and 297-D4

Three dimensional (3D) structures of 144-A8 and 297-D4 were constructed from homology modeling with the latest version of Modeller program [19], using the X-ray crystal structure of IGG1 antibody (PDB ID: 32C2) as the structural template [20]. Because sequence identity between the target and template proteins amounts to higher than 80%, good structural models for both antibodies could be obtained in the homology modeling. The structure of epitope was extracted from the X-ray crystal structure of BAP31 (PDB ID: 4JZL) [21], which comprises ten amino acids in the sequence of QVLAMRKQSE. Gasteiger-Marsilli atomic charges were assigned for all receptor and ligand atoms to calculate the electrostatic interaction in the antibody-antigen complexes [22]. Docking simulations were then carried out with the AutoDock program [23] to obtain the binding mode of epitope in CDRs of 144-A8 and 297-D4. Of the 20 conformations of epitope generated in docking simulations, those clustered together had similar binding modes, differing by less than 1.5 Å in positional root-mean-square deviation. The most stable binding configuration in the top-ranked cluster was selected as the final structural model for antigen-antibody complex.

## Results and Discussion

### Two MAbs 144-A8 and 297-D4 show different binding specificities to some cells

**Fig 1:**
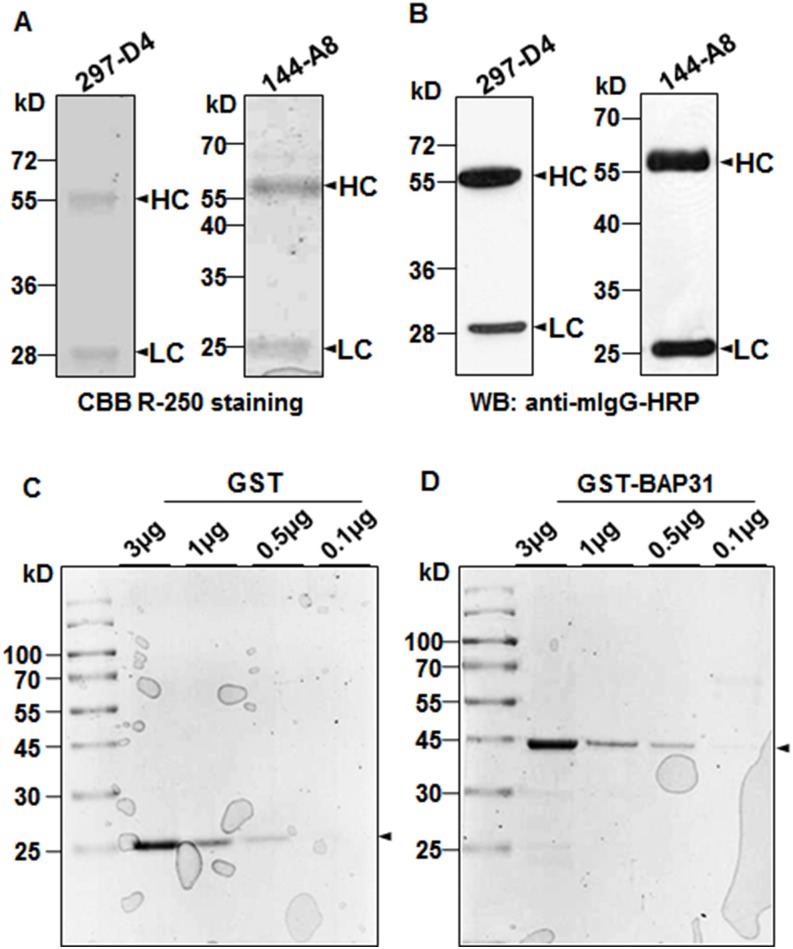
Purification of MAbs and GST-fusion proteins. (A) Purified antibodies were subjected to 12% SDS-PAGE and stained with Coomassie Brilliant Blue R-250. (B) Western blot analysis of the purified antibodies with HRP-conjugated anti-mouse IgG antibody. (C, D) Purification of GST and GST-BAP31 fusion proteins. GST (C) and GST-BAP31 (D) fusion proteins were purified using glutathione Sepharose beads from bacterial lysates. The purified proteins were subjected to 12% SDS-PAGE gel and stained using Coomassie Brilliant Blue R-250. Arrowheads indicate GST or GST-BAP31 proteins.

In the previous study, we found that the two MAbs, 297-D4 and 144-A8, recognize a linear epitope (residues 208–217) on the C-terminal domain of BAP31 [14, 15]. The epitope is composed of 10 continuous amino acids (QVLAMRKQSE) and exposed on the cell surface [15]. When cell surface expression of BAP31 was monitored on various cells by the two MAbs, 144-A8 exhibited an additional binding reactivity to A172, MDA-MB435 and NCI-H522 cells (S1 Fig and S1 Table), suggesting that the amino acid sequences of 144-A8 may be different from those of 297-D4, although they recognize the same epitope. Western blot analysis of the purified antibodies also showed that the molecular weight of the light chain of 297-D4 was slightly larger than that of 144-A8 (Fig 1A and 1B), suggesting again that they have different amino acid sequences.

### 144-A8 shows higher antigen binding capacity than 297-D4

**Fig 2:**
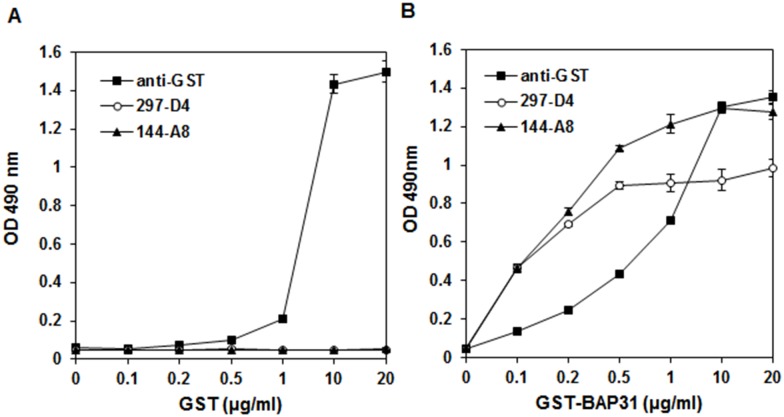
Comparison of antigen binding capacities of 297-D4 and 144-A8 antibodies depending on antigen dosage. Increasing concentrations of GST (A) and GST-BAP31 (B) were coated and incubated with the indicated antibodies. The binding activities of the antibodies are expressed as OD490. Error bars represent standard deviations of the means.

Previously, we generated a series of GST-fused BAP31 mutant proteins, in which BAP31 was serially deleted at the C- terminus, and found that 297-D4 and 144-A8 recognize residues 208–217 on the C-terminal domain [15]. To stably express and purify BAP31 recombinant antigen, the C-terminal domain (the residues 124–246), a transmembrane domain-free BAP31 fragment, was fused to the GST gene, and the GST-BAP31 fusion protein was expressed and purified. As a control, the GST protein was also purified. The purified GST and GST-BAP31 were observed around 25 kDa and 44 kDa on 12% SDS-PAGE, respectively (Fig 1C and 1D). To examine the binding specificities of 297-D4 and 144-A8 to the purified proteins, ELISA plates containing increasing concentrations of the purified GST proteins were tested with the same amounts of antibodies in an indirect ELISA. Both 297-D4 and 144-A8 only bound to the purified GST-BAP31 while anti-GST antibody bound to both the purified GST-BAP31 and GST proteins (Fig 2A and 2B), indicating that both 297-D4 and 144-A8 had binding specificity to the recombinant BAP31 antigen. The antigen binding capacity of 297-D4 was almost saturated at approximately 0.5 μg/ml of antigen while that of 144-A8 was saturated at concentrations higher than 20 μg/ml of antigen. Thus, 144-A8 showed higher antigen binding capacity at the same concentration of antigen than 297-D4.

### 144-A8 shows higher binding affinity than 297-D4

**Fig 3:**
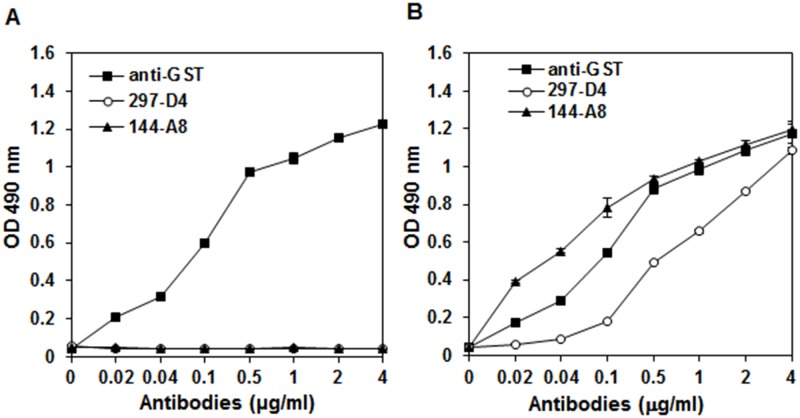
Comparison of antigen binding capacities of 297-D4 and 144-A8 antibodies depending on concentration of antibodies. GST (A) and GST-BAP31 (B) were coated and incubated with increasing concentrations of anti-GST, 297-D4 and 144-A8 antibodies. The binding activities of the antibodies are expressed as OD490. Error bars represent standard deviations of the means.

**Fig 4:**
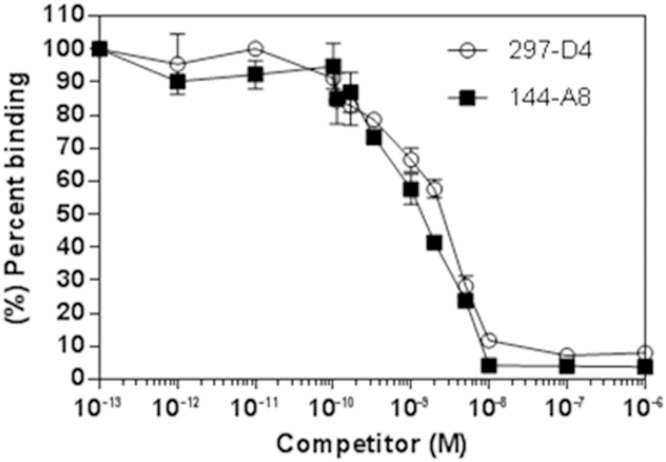
Affinity determination of 144-A8 and 297-D4 using competitive ELISA. Increasing concentrations of GST-BAP31 were pre-incubated with 297-D4 and 144-A8 in the solution first, and free antibodies were further incubated with coated GST-BAP31. The binding activities of free antibodies are expressed as % binding. Each experimental point is the mean of three independent experiments performed in triplicates.

In order to compare the affinity of 144-A8 with 297-D4, ELISA plates coated with 20 μg/ml of purified GST or GST-BAP31 were incubated with increasing concentrations of antibodies in an indirect ELISA (Fig 3A and 3B). Again, both 297-D4 and 144-A8 only bound to the GST-BAP31 protein while anti-GST antibody bound to both the GST-BAP31 and GST proteins (Fig 3A and 3B). When the binding capacities of 297-D4 and 144-A8 to the GST-BAP31 protein were compared, the GST-BAP31 protein was readily recognized by 144-A8 even at concentrations lower than 0.02 μg/ml, while it was recognized by 297-D4 at higher concentrations than 0.1 μg/ml, suggesting that 144-A8 has higher affinity to the GST-BAP31 protein than 297-D4. To compare the binding affinities of two antibodies more precisely, we determined the affinity of each antibody towards the GST-BAP31 protein by competitive ELISA (Fig 4). The affinities of 144-A8 and 297-D4 towards the GST-BAP31 protein were approximately 6.81 x 10^8^ M^-1^ and 4.43 x 10^8^ M^-1^, respectively. These data indicate that the antibody affinity of 144-A8 is about 1.54-fold higher than 297-D4.

### Cloning and sequencing the variable regions of heavy and light chains of 297-D4 and 144-A8

**Fig 5:**
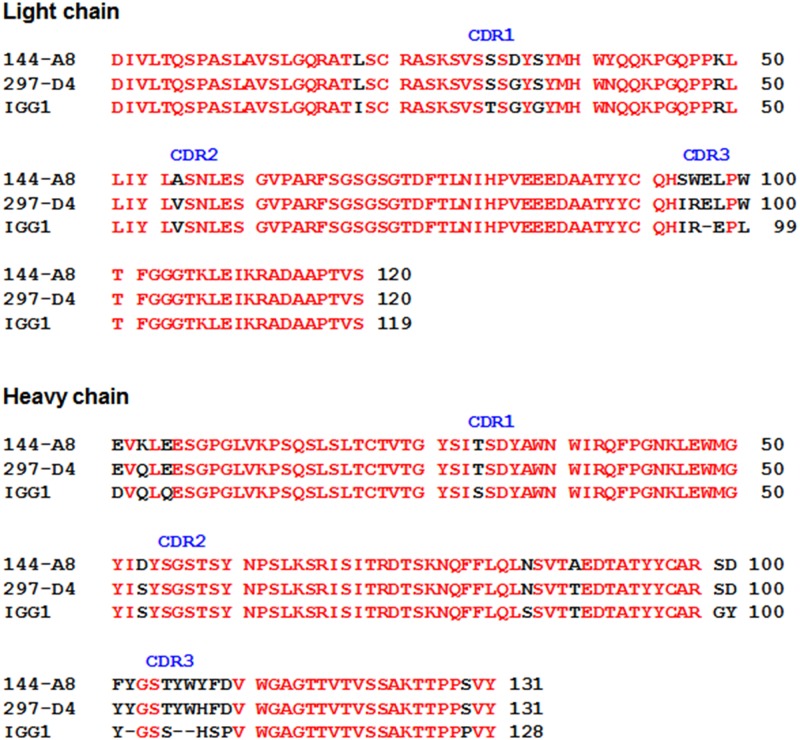
Alignment of the amino acid sequence of the variable regions of 297-D4 and 144-A8 with respect to a homologous antibody (IGG1) for which the X-ray crystal structure is known. The identical amino acids among the three antibodies are indicated in red.

Two MAbs 144-A8 and 297-D4 recognize the same epitope composed of 10 amino acids (QVLAMRKQSE) on BAP31 [15], but affinity measurement showed that they had different binding affinities towards the BAP31 protein. Therefore, we examined the sequence difference between the two MAbs by cDNA sequencing of their light and heavy chain variable regions. The amino acid sequences of light and heavy chain variable regions of two MAbs were compared to the database of mouse immunoglobulin sequences by using VBASE2 (www.vbase2.org) [24] (Fig 5). Sequence analysis showed that both MAbs belonged to the same heavy chain (Igh-V3660 VH3) and light chain subgroup (IGKV21), and each of the light and heavy chains displayed almost the same amino acid sequences with few exceptions. Two MAbs 144-A8 and 297-D4 were independently established [15]. Therefore, it is possible to speculate that the lymphocyte clones with the same germline sequences were selected against the same BAP31 epitope, but they underwent different point mutations under different microenvironments during affinity maturation. A total of 4 amino acid changes were observed in the frameworks of 144-A8 light and heavy chains, as compared to those of 297-D4 light and heavy chains. The observed variations of 144-A8 were not prominent, as compared to the cognate amino acids of 297-D4. Gln-3 (Q3) of 297-D4 heavy chain was changed to Lys-3 (K3) of 144-A8 (Fig 5), and the alteration seemed to be prominent because of charge change. However, the alteration occurred in the N-terminal part of framework 1, and the mutation does not affect antigen binding [25].

### Docking and molecular dynamics simulations of the BAP31 epitope in CDRs of 144-A8 and 297-D4

**Fig 6:**
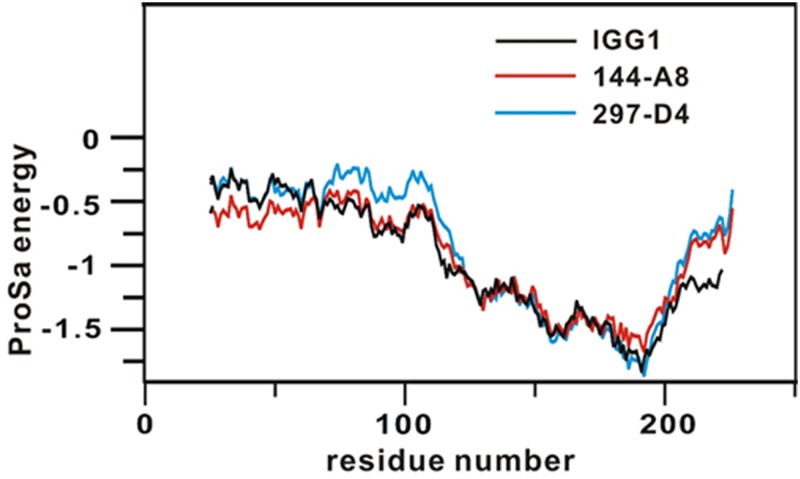
Comparative view of the ProSa energy profiles for the homology-modeled structures of 297-D4 and 144-A8 in comparison with that of the structural template (IGG1). For convenience, the amino acid sequences of light and heavy chains are combined and the combined amino acids are renumbered from 1 instead of retaining the original numbers.

CDRs are believed to be responsible for recognition of the epitope [25]. Seven amino acid changes were found in the CDRs of 144-A8, as compared to those of 297-D4 (Fig 5). Structures of 144-A8 and 297-D4 were obtained with homology modeling using the X-ray crystal structure of Fab fragment (IGG1) to cytochrome P450 aromatase (PDB ID: 32C2) as the structural template [20]. The structures of the gap regions in CDR3 were optimized from a randomly distorted structure bridging the two anchoring regions as implemented in MODELLER. To validate the homology-modeled structures of 144-A8 and 297-D4, their conformational energies were calculated with the ProSa 2003 program [26]. This program has been widely used to judge the quality of a protein fold by calculating the knowledge-based mean fields. As shown in Fig 6, the ProSa energies of the predicted structures of 144-A8 and 297-D4 antibodies are maintained negative for most amino acid residues although they remain higher than IGG1 in C-terminal regions of light and heavy chains. Due to these good energetic features, 3-D structures of 144-A8 and 297-D4 predicted using the X-ray crystal structure of IGG1 as the template was selected for the subsequent docking simulations with the epitope.

**Fig 7:**
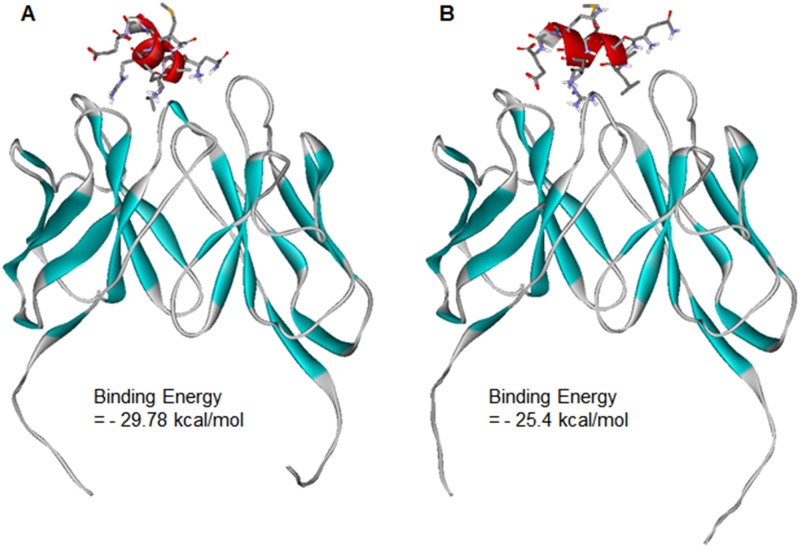
Docked poses of the antigen epitope in the complementarity determining regions of (A) 144-A8 and (B) 297-D4. The β-strands of the antibodies and the α-helices of the epitope are shown in cyan and red, respectively. Carbone atoms of epitope are indicated in grey.

Docking simulations of BAP31 epitope were carried out in CDRs of 144-A8 and 297-D4 to address the differences in binding affinities and in binding patterns, which are compared in Fig 7. Overall, the epitope appears to reside in closer proximity to the CDRs of 144-A8 than to those of 297-D4. The calculated binding free energy of 144-A8-epitope complex was 4.4 kcal/mol lower than that of 297-D4-epitope complex, which was consistent with the higher binding affinity of 144-A8 than 297-D4. To examine the possibility of binding of the epitope in the allosteric sites, we conducted additional docking simulations with extended 3D grid maps that included the whole antibody structures. However, no peripheral binding site was identified in which the epitope could be accommodated with the negative free energy of binding. This supports the possibility that QVLAMRKQSE would be the epitope of BAP31 antigen.

**Fig 8:**
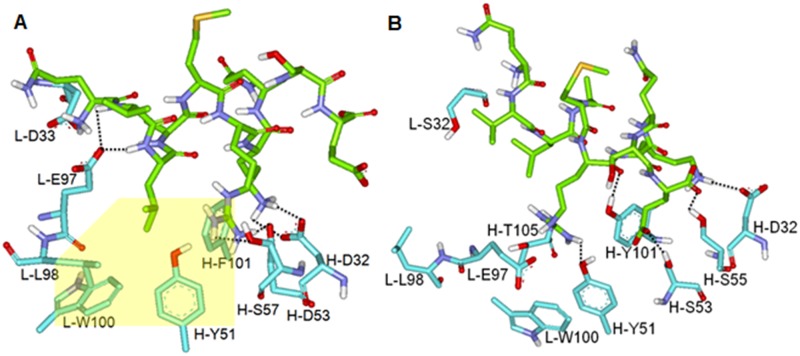
Calculated binding mode of epitope in complementarity determining region of (B) 144-A8 and (B) 297-D4. Carbon atoms of epitope and antibody are indicated in green and cyan, respectively. Dotted lines and yellow box indicate the hydrogen bonds and the hydrophobic interactions, respectively.

To address the detailed interactions responsible for the stabilization of BAP31 epitope in the CDRs of the antibodies, the lowest-energy conformations of the epitope in CDRs of 144-A8 and 297-D4 are compared in Fig 8. Consistent with a large difference in binding energies, the two antibody-antigen complexes reveal the different patterns for the establishment of the hydrogen bonds. A total of six and five hydrogen bonds were observed at the interfaces of 144-A8-epitope and 297-D4-epitope complexes, respectively, with different CDR residues being involved in the hydrogen bonds. The epitope appears to be stabilized in CDRs of 144-A8 through the hydrogen bonds with the side-chain carboxylate groups of light and heavy chains (L-Glu97, H-Asp32, and H-Asp53) as well as with the side-chain hydroxyl moiety of H-Ser55. On the other hand, the side-chain hydroxyl groups in the heavy chain of 297-D4 (H-Tyr51, H-Ser53, H-Ser55, and H-Tyr101) were involved in the complexation with the epitope together with the carboxylate moiety of H-Asp32. The hydrogen bond interactions are thus quite different between the two antibody-epitope complexes although both complexes include the same epitope. This can be understood in the context of the difference in amino acid sequences in the CDR3 region (Fig 5) between the two antibodies. Judging from the involvement of the more charged residues of 144-A8 than 297-D4 in the formation of hydrogen bonds, the higher binding affinity of the former than the latter can be attributed to the strengthening of the hydrogen bond interactions in the antibody-epitope complex.

Hydrophobic interactions appear to be established also in different fashion in the two antibody-epitope complexes. Most remarkably, the leucine side chain of epitope was accommodated in a hydrophobic pocket, comprising the side chains of nonpolar residues in the light chain (L-Leu98 and L-Trp100) and those in the heavy chain (H-Tyr51 and H-Phe101) of 144-A8. Due to the substitution of H-Phe101 for H-Tyr101 in 297-D4, on the other hand, the leucine side chain of epitope is exposed to the bulk solvent in 297-D4-epitope complex. Thus, the hydrophobic interaction appears to be an additional interaction found in the 144-A8-epitope complex. The replacement of H-Tyr with H-Phe at residue 101 could thus have the effect of facilitating the interactions between 144-A8 and the epitope. Indeed, the epitope appears to reside in closer proximity to the CDRs of 144-A8 than to those of 297-D4 (Fig 7) because its leucine side chain can be accommodated only in the hydrophobic binding pocket in 144-A8-epitope complex. Besides the strengthening of the hydrogen bond interactions with the epitope, the appearance of the additional hydrophobic interactions may be also invoked to explain the higher binding affinity in the 144-A8-epitope complex than in the 297-D4-epitope counterpart.

**Fig 9:**
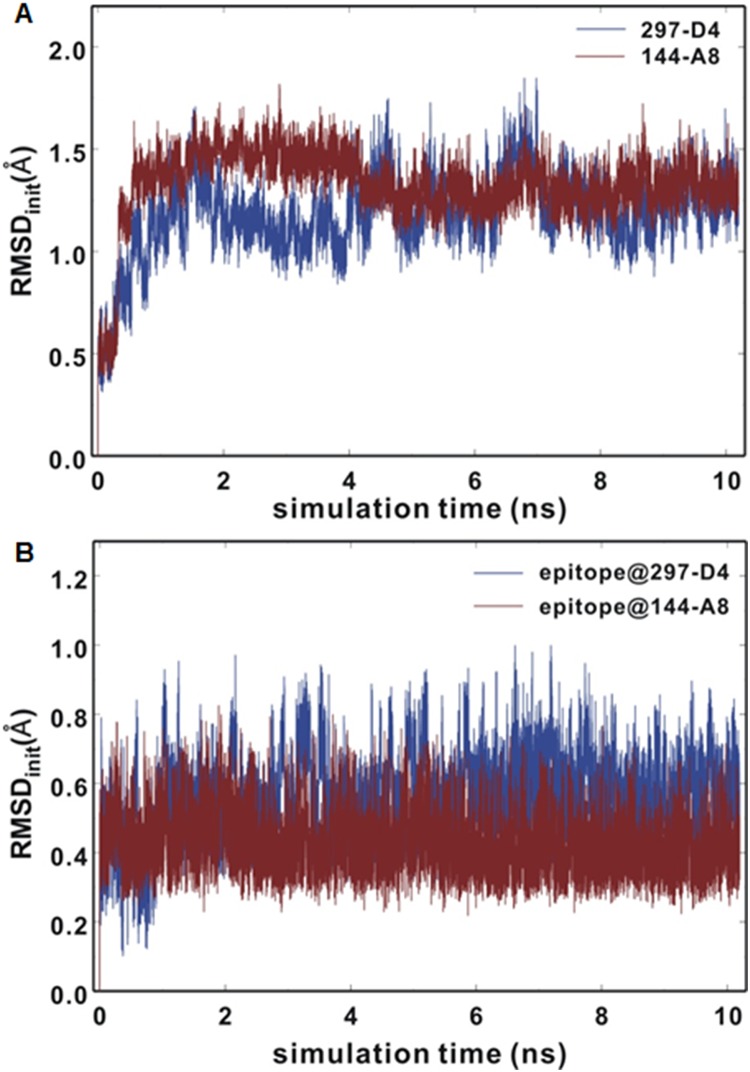
Time evolutions of RMSD_init_ values of (A) antibody backbone C_α_ atoms and (B) heavy atoms of the epitope from the starting structures prepared from docking simulations.

To address the structural flexibilities of epitope-antibody complexes, we carried out 10.2 nanosecond molecular dynamics (MD) simulations of 144-A8 and 297-D4 antibodies in complex with the epitope using the latest version of AMBER program with the explicit solvent model including 10266 water molecules [27]. Fig 9 shows the time evolutions of the root mean square deviations (RMSD_init_) for backbone C_α_ atoms of the antibodies and those for the heavy atoms of the epitope with respect to the starting structures prepared from the precedent docking simulations. These RMSD_init_ data were calculated from the MD trajectory snapshots to examine the dynamic stabilities of both antibody-epitope complexes in aqueous solution. The RMSD_init_ values remain within 2.0 Å during the entire course of simulation in both cases with a convergent behavior with respect to simulation time. This implies that protein conformations of the antibodies would be maintained stable in interacting with the epitope. The positional shifts of the epitope in the CDR regions seem to be insignificant when compared to the conformational changes of the antibodies because the RMSD_init_ values for the heavy atoms of the epitope fall within 1 Å and remain lower than those of C_α_ atoms of the antibodies. Consistent with the higher binding affinity of the epitope for 144-A8 than for 297-D4 antibody, the RMSD_init_ values of the epitope in complex with the former appear to be lower than those in complex with the latter for the majority of simulation time. Thus, both docking and MD simulation results support the experimental implication that the epitope binds to 144-A8 in preference to 297-D4.

In summary, the binding specificity and affinity of 144-A8 were broader and higher than those of 297-D4, although two MAbs recognized the same epitope composed of 10 linear amino acids. There were 7 amino acid differences between the CDRs of two MAbs. Molecular modeling studies revealed that the epitope appeared to reside in closer proximity to the CDRs of 144-A8 than to those of 297-D4 with the stronger hydrogen bond interactions with the former than the latter. The substitution of H-Tyr101 for H-Phe101 in 144-A8 appeared to induce an additional hydrophobic interaction between the Leu residue of epitope and the paratope of 144-A8, explaining the higher affinity of 144-A8 than that of 297-D4. Thus, the alterations of amino acids in the CDRs of 144-A8 appeared to be responsible for the generation of different hydrogen bonds and hydrophobic interactions, which characterizes the different binding specificity and affinity of 144-A8. This study provides important molecular insights into how the binding specificities and affinities of antibodies evolve with the same epitope during affinity maturation.

## Supporting Information

S1 Fig Flow cytometric analysis of various cells with 297-D4 or 144-A8 antibodies. (TIF)

S1 Table Binding profiles of 297-D4 and 144-A8 antibodies to various cells. (DOCX)
