# Supplementary figures and images for: Molecular Characterization of Two Monoclonal Antibodies against the Same Epitope on B-Cell Receptor Associated Protein 31
Source: PLoS One. 2016 Dec 1;11(12):e0167527. doi: 10.1371/journal.pone.0167527 (PMC5131989; doi:10.1371/journal.pone.0167527)

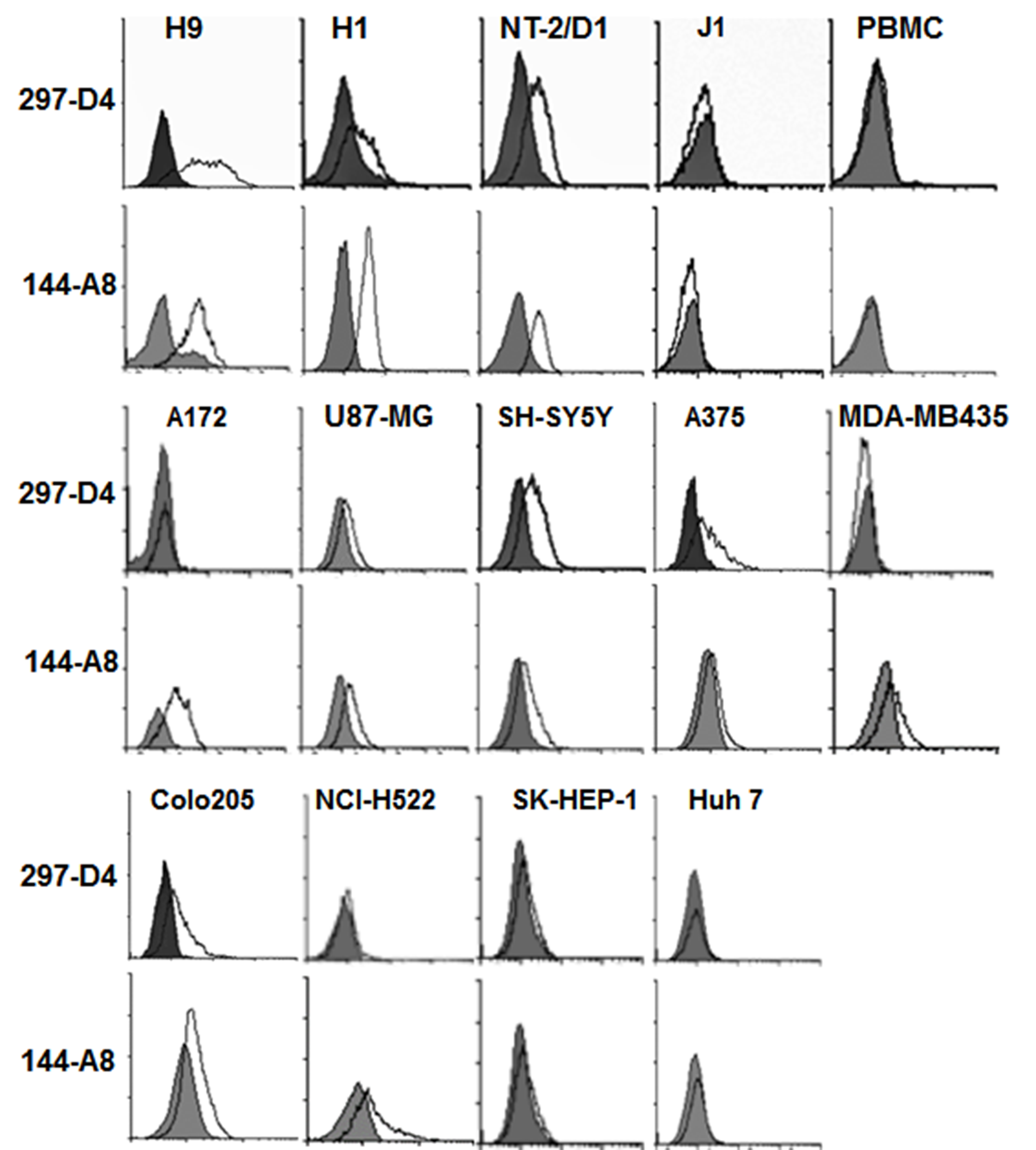

Supplement: S1 Fig — (TIF) [file pone.0167527.s001.tif]
