# Supplementary material for: Molecular Characterization of Two Monoclonal Antibodies against the Same Epitope on B-Cell Receptor Associated Protein 31
Source: PLoS One. 2016 Dec 1;11(12):e0167527. doi: 10.1371/journal.pone.0167527 (PMC5131989; doi:10.1371/journal.pone.0167527)
Supplement: S1 Table — (DOCX) [file pone.0167527.s002.docx]

| Name | Cells | 297-D4 | 144-A8 |
| --- | --- | --- | --- |
| H9 | Human embryonic stem cell | +++ | +++ |
| H1 | Human embryonic stem cell | + | ++ |
| NT-2/D1 | Human embryonal carcinom | ++ | ++ |
| NCCIT |  | +++ | +++ |
| mES(J1) | Mouse embryonic stem cell | - | - |
| PBMC | Peripheral blood monocytes | - | - |
| A172 | Glioblastoma, brain | - | ++ |
| U-87MG | Glioblastoma, brain | + | + |
| SH-SY5Y | Neuroblastoma, brain | + | + |
| A375 | Malignant melanoma, skin | + | + |
| MDA-MB435 | Ductal carcinoma, breast | - | + |
| Colo-205 | Adenocarcinoma, colon | + | + |
| NCI-H522 | Adenocarcinoma, lung(NSCLC) | - | + |
| SK-HEP-1 | Adenocarcinoma, liver | - | - |
| Huh7 | Hepatocellular carcinoma, liver | - | - |

**S1 Table. Binding profiles of 297-D4 and 144-A8 antibodies to various cells.**

+++, strong binding; ++, medium binding; +, weakly binding; -, no binding.
